# Overweight Leads to an Increase in Vitamin E Absorption and Status in Mice

**DOI:** 10.1002/mnfr.202400509

**Published:** 2024-11-16

**Authors:** Katherine Alvarado‐Ramos, Ángela Bravo‐Núñez, Donato Vairo, Charlotte Sabran, Jean‐François Landrier, Emmanuelle Reboul

**Affiliations:** ^1^ Aix‐Marseille Université, INRAE, INSERM, C2VN Marseille France; ^2^ Universidad de Valladolid Palencia 34004 Spain

**Keywords:** adipose tissue, intestine, lipid transporters, liver, obesity, tocopherol

## Abstract

**Scope:**

This study investigates whether vitamin E (VE) deficiency in subjects with obesity could, at least partly, be due to a defect in VE intestinal absorption.

**Methods and results:**

Mice follow either a high‐fat (HF) or a control (CTL) diet for 12 weeks. The study evaluates their VE status, the expression of genes encoding proteins involved in lipid and fat‐soluble vitamin intestinal absorption, and VE absorption using a γ‐tocopherol‐rich emulsion. HF mice have a weight (+23.0%) and an adiposity index (AI, +157.0) superior to CTL mice (*p* < 0.05). α‐Tocopherol concentrations are higher in both plasma (+45.0%) and liver (+116.9%) of HF mice compared to CTL mice (*p* < 0.05). α‐Tocopherol concentration in the adipose tissue of HF mice is higher than that of CTL mice after correction by the AI (+72.4%, *p* < 0.05). No difference is found in the expression of genes coding for proteins involved in intestinal lipid metabolism in fasting mice. After force‐feeding, γ‐tocopherol plasma concentration is higher in HF mice compared to CTL mice (+181.5% at 1.5 h after force‐feeding, *p* < 0.05).

**Conclusion:**

HF mice display higher status and more efficient absorption of VE than CTL mice. VE absorption is thus likely not impaired in the early stages of obesity.

## Graphical abstract

Mice fed a high‐fat diet for 12 weeks showed higher vitamin E status and more efficient absorption than control mice.

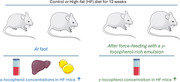

## 1 Introduction

About 38% of the World population now present a BMI >25 kg m^−2^ according to World Obesity Data.^[^ ^1^ ^]^ People with overweight and obesity frequently present decreased levels of circulating carotenoids, minerals, and vitamins such as vitamin A, B_9_, D, E, and C.^[^ ^2^, ^3^, ^4^, ^5^, ^6^, ^7^, ^8^ ^]^ The causes of these deficiencies remain unclear. Deficiency levels depend on the vitamin. For instance, compared to normal plasma levels, vitamin decreases range from −16% for vitamin A to −40% for vitamin E (VE) or −80% for vitamin C.^[^ ^9^ ^]^ This phenomenon is called the obesity paradox: people with obesity have higher needs due to high body weight, but despite higher dietary intake, these needs are not met given their circulating vitamin levels.^[^ ^4^ ^]^

VE represents a group of fat‐soluble compounds gathering four tocopherols and four tocotrienols. α‐Tocopherol is the vitamer with the highest vitamin activity. VE plays important roles in preventing neurodegenerative diseases^[^ ^10^ ^]^ and ocular pathologies^[^ ^11^ ^]^, as well as in reducing the oxidation of low‐density lipoproteins.^[^ ^12^ ^]^ In mice with obesity, α‐tocopherol was shown to reduce adipose tissue (AT) fibrosis, inflammation, and oxidative stress.^[^ ^13^ ^]^

VE main dietary sources are oils such as sunflower oil, nuts, and vegetables including pumpkin, red bell pepper, and avocado.^[^ ^14^ ^]^ VE follows the fate of lipids during the digestion process. VE esters, if any, are hydrolyzed by cholesterol esterase in the duodenum. Free VE is ultimately included in mixed micelles with other lipid digestion products and can then approach the brush border through the unstirred water layer and the glycocalyx. At the apical side of enterocytes, three cholesterol transporters have been involved in VE uptake: SR‐BI, CD36, and NPC1L1.^[^ ^15^ ^]^ Once in enterocytes, free VE is packed into chylomicrons and secreted to the lymph before joining the blood compartment. A fraction of VE may also be secreted by ABCA1 via an ApoAI‐dependent pathway.^[^ ^15^ ^]^ VE main storage tissues are the liver and the AT. VE can be metabolized by the CYP4F2 into carboxyethylchromanes and be excreted by the biliary and urinary ways.^[^ ^16^, ^17^ ^]^

VE deficiency observed in patients with obesity can have different origins: i) a decreased intake, ii) a defect in absorption, iii) an over‐metabolization, or iv) an over‐excretion.

In this work, we challenged the second possibility. To this aim, we assessed the α‐tocopherol status of mice that received either a control (CTL) diet of a high‐fat (HF) diet for 3 months. Mice were also force‐fed with γ‐tocopherol in an oil‐in‐water emulsion to evaluate VE postprandial response.

## 2 Experimental Section

### 2.1 Chemicals

α‐ and γ‐tocopherols were purchased from Sigma–Aldrich (Saint‐Quentin‐Fallavier, France). Tocol (internal standard for HPLC analyses) was purchased from Lara Spiral (Couternon, France). Triton1339 (Tyloxalapol) was purchased from Thermo Fischer (Thermo Fisher Scientific, Villebon sur Yvette, France). Lesieur Isio4 oil was purchased from a local supermarket (Casino, France). Phosphate‐buffered saline (PBS) was purchased from Life Technologies (Illkirch, France). TRIzol reagent was purchased from Euromedex (Souffelweyersheim, France). All solvents were HPLC grade and obtained from Carlo Erba (Peypin, France).

### 2.2 Mice Preparation and Diet

The protocol was authorized by The French Ministry of Research and the ethics committee of Aix‐Marseille University under the number APAFIS #34938‐2022031410115497 v5. Forty C57BL6J male mice were purchased from Janvier Labs. The mice arrived at 6 weeks of age and were identified by ear punching. Immediately after arrival, the mice underwent a 7‐day acclimatization period under controlled temperature, humidity, and lighting conditions. Subsequently, the mice were divided into two groups according to the experimental diet. The groups were supplied with different food pellets ad libitum. The CTL group received a standard diet providing 17.4% of the energy from lipids and 87 IU kg^−1^ α‐tocopherol (Safe® AIN93G), while the other group (HF) was fed an HF diet providing 46% of energy from lipids and 104 IU kg^−1^ α‐tocopherol (Safe^®^ 251 HF). CTL and HF diet compositions are given in Supporting Information Table S1.

Mouse weight was measured every week for 12 weeks. Mouse food intake was determined at the cage level. At 11 weeks, a blood sample was taken from the tail under anesthesia (isoflurane) to establish a baseline for all mice. Blood was collected using heparinized capillaries, centrifuged in situ, and plasma was collected and stored at −80°C until analysis.

**FIGURE 1:**
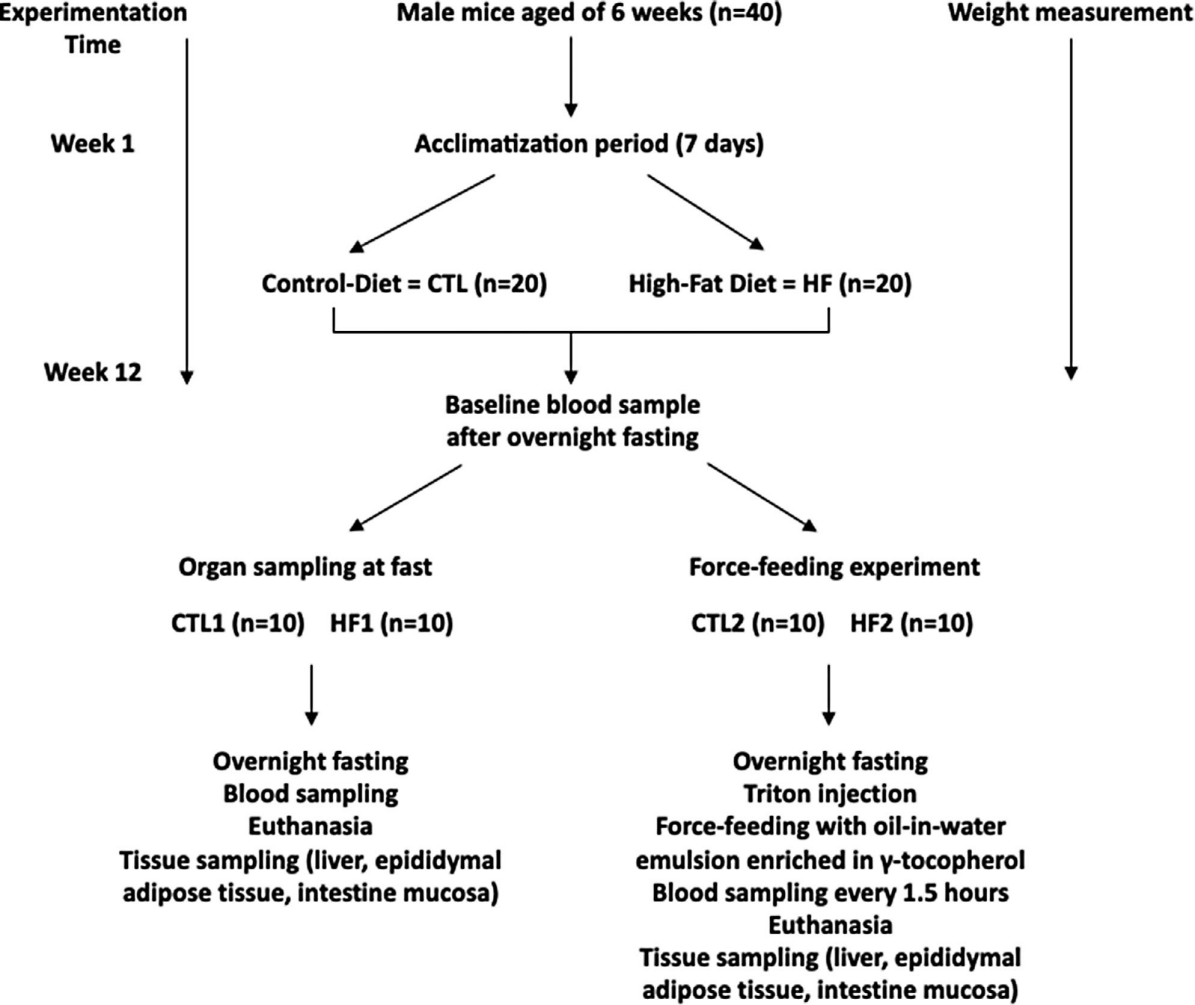
Study flow chart. After 12 weeks of feeding with a control (CTL) or high fat (HF) diet, mice were divided in four groups: CTL1, CTL2, HF1, and HF2.

At 12 weeks, the mice were subdivided into four groups: CTL 1 (*n* = 10), CTL 2 (*n* = 10), HF 1 (*n* = 10), HF 2 (*n* = 10), and fasted overnight before experiments (**Figure** **1**).

### 2.3 Tissue Sampling

Fasting mice from CTL1 and HF1 groups were euthanized by cervical dislocation under anesthesia (isoflurane), and an intracardiac blood sample was collected using preheparinized tubes. Plasma was collected after centrifugation and stored at −80°C until analysis. Organs (liver, epididymal AT, and intestinal mucosa) were promptly harvested. The intestines were washed with PBS and divided on ice into five parts equivalent to the duodenum, proximal jejunum, median jejunum, distal jejunum, and ileum. The mucosa was carefully collected. Organs, mucosa, and plasma were immediately snap‐frozen in liquid nitrogen after collection and stored at −80°C until analysis. All samples were collected with hygienic and sterile measures free of RNases.

### 2.4 Postprandial Experiment

CTL2 and HF2 groups followed a postprandial experimental protocol. Fasting mice were intravenously injected with Triton 1339 to inhibit lipoprotein lipase. The Triton dose was adjusted to mouse weight (0.5 mg g^−1^). After 9 min, mice were force‐fed with an oil‐in‐water emulsion (1/2, v/v) enriched in γ‐tocopherol. The emulsion solution was prepared as follows: 40 µL of a solution of γ‐tocopherol (12.5 mg mL^−1^ in ethanol) was mixed with 250 µL of a solution of lysophosphatidylcholine (4% in ethanol) for stabilization. Ethanol was evaporated under nitrogen and 100 µL of Isio_4_ was added. Two hundred microliter of water was then added to the mixture and sonified in an ice bath (Branson 3510) for 10 min. The quantity of the emulsion given to each mouse was also adjusted to mouse weight (10 µL g^−1^). Blood sampling was performed by either submandibular or retroorbital puncture under anesthesia every 1.5 h for 6 h. One mouse appeared to be in pain during the protocol and was immediately euthanized. Blood was collected using preheparinized tubes. Plasma was collected after centrifugation and stored at −80°C until analysis. After cervical dislocation, organs were harvested as described above. The intestine was washed, divided in five parts, and stored intact. All organs were immediately snap‐frozen in liquid nitrogen and stored at −80°C until analysis.

### 2.5 Vitamin E Assay

#### 2.5.1 Plasma

α‐Tocopherol and γ‐tocopherol were extracted from 20‐50 µL of plasma. 450–480 µL of ultrapure water and 500 µL of tocol in ethanol (internal standard) were added to the samples. The mixtures were extracted twice with 2 mL of hexane, shaken for 10 min, and centrifuged for 10 min at 2000 × *g* at 4 °C. The upper phases were pulled and dried under nitrogen. The dried residues were resuspended in 200 µL of methanol and placed in vials for analysis by HPLC. HPLC analysis was performed on a Shimadzu separation module (LC‐20ADSP HPLC Pumps and SIL‐20CHT Autosampler, Shimadzu, Champs‐sur‐Marne, France) coupled with a Themofisher fluorescence detector (Thermo Fischer Scientific, excitation at 292 nm emission at 325 nm). The mobile phase was 100% methanol with a flow rate of 1.5 mL min^−1^. The C18 column was kept at 40 °C. A volume of 50‐150 µL was injected. The identification of the vitamin was done with Chromeleon 7.2 (Thermo Fischer Scientific). Quantification was performed by comparing peak areas to standard curves.

#### 2.5.2 Liver and Intestines

Both liver and intestines were prepared according to a previously described protocol.^[^ ^18^ ^]^ Intestine samples and the right lobe of the liver were ground in 1 mL of PBS using a Retsch (Retsch, Erangy sur Oise, France) at 30 mvt s^−1^. The homogenates were stored at −80 °C until analysis.

Quantification of α‐tocopherol and γ‐tocopherol in the liver and intestines was performed after a Blight & Dyer extraction protocol.^[^ ^19^ ^]^ The extracted lipids were resuspended in 200 µL of methanol and placed in vials for HPLC analysis as described in section 2.5.1.

#### 2.5.3 Adipose Tissue

To quantify the α‐tocopherol and γ‐tocopherol contents of AT, the study used a protocol adapted from Cuerq et al^[^ ^20^ ^]^. Briefly, 32.5 mg of AT was weighed and placed in a 2 mL Eppendorf® tube. Then, 200 µL of ethanol and 200 µL of tocol in ethanol (internal standard) along with two grinding balls were added. The mixture was ground in a Retsch for 5 min at 9 mvt s^−1^. The mixture was extracted twice with 200 µL ultrapure water and 500 µL hexane. The upper phases were pulled and dried under nitrogen. The dried residues were resuspended in 200 µL methanol and placed in vials for HPLC analysis as described in section 2.5.1.

### 2.6 Cholesterol and Triglyceride Assays

Cholesterol and triglycerides were quantified in mouse plasma, as well as in the liver and intestines. For the liver and intestines, an extraction was performed from the previously described homogenate using the Blight & Dyer protocol. The lipids were resuspended in 400 µL of isopropanol and stored at ‐20 °C until analysis. Measurements for both plasma and organ lipids were made using Biolabo kits (Maisy, France) according to the manufacturer's instruction.

### 2.7 Real‐Time PCR

A semi‐quantitative qPCR or reverse transcription PCR was performed to quantify the expression of genes encoding proteins involved in the absorption of fat‐soluble vitamins and lipids. The following genes were measured: *Scarb1, Cd36, Npc1l1, Abcg5, Abcg8, Abca1, Abcb1b, Apo48, Mttp*, and *18S* (reporter gene).

#### 2.7.1 RNA Extraction and Real‐Time PCR

Total RNA from the intestinal mucosa of fasting mice was extracted with TRIzol reagent as previously described^[^ ^21^ ^]^ and stored at −20°C until use. For the real‐time PCR, samples containing 1 µg µL^−1^ of RNA were diluted in 9 µL of RNAse‐free water containing an intercalant agent (SYBR Green, Thermo Fisher Scientific) and specific primers (Eurogentec, Angers, France, Supporting Information Table S2). Gene expression was measured using a Thermocycler Light Cycler 480 (Roche, Basel, Switzerland). Each sample was analyzed in duplicate, and 18S rRNA was used as internal CTL.

### 2.8 Adiposity Index Calculation

Adiposity index (AI) was calculated as follows:

(1) $$\mathrm{AI}\;=\frac{\mathrm{Epididymal}\;\mathrm{adipose}\;\mathrm{tissue}\;\left(g\right)}{\mathrm{Mouse}\;\mathrm{weight}\;\mathrm{the}\;\mathrm{day}\;\mathrm{of}\;\mathrm{the}\;\mathrm{experiment}\;\left(g\right)}\;\;\times 100$$

### 2.9 Statistical Analysis

All data were analyzed using RStudio version 4.3.2. Results are means ± SEM. For the postprandial experiment, we used the trapezoidal approximation to obtain the AUC of postprandial lipid and VE concentrations. For qPCR results, gene expression was standardized with the CTL group as 100%. After verifying the nature of data, homoscedasticity, and normal distribution, differences between groups were examined with the Kruskal–Wallis test and if necessary a post hoc test Dunn–Nemenyi. A *p* value < 0.05 was considered significant.

## 3 Results

### 3.1 High‐Fat Diet Induces a Moderate Obesity in Mice Compared to the Control Diet

**FIGURE 2:**
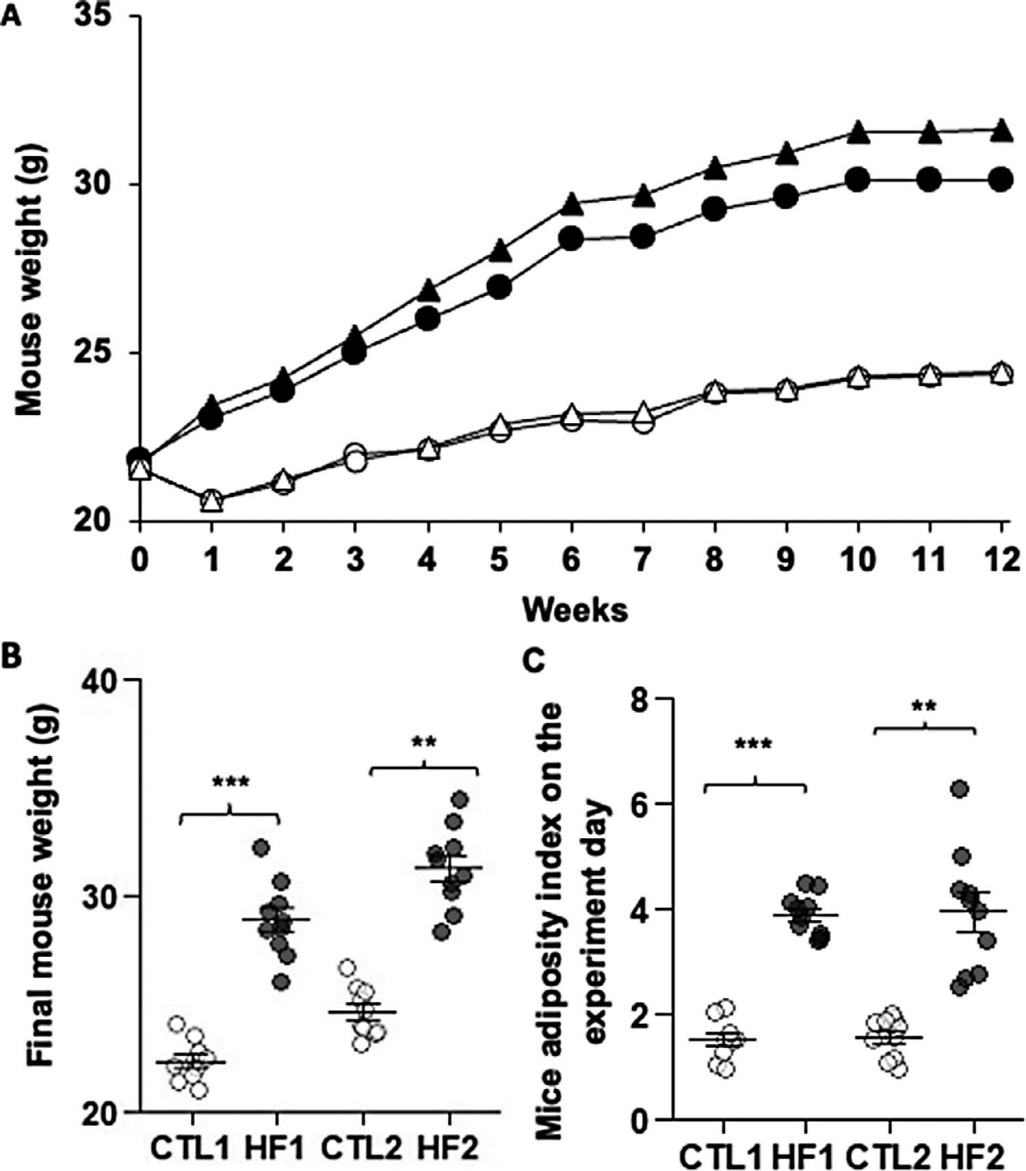
Mouse weight and adiposity index. A) Mouse weight measurement during 12 weeks. B) Mouse final body weight the day of the experiment. C) The adiposity index calculated as the ratio between the weight of epididymal adipose tissue and mouse body weight. CTL groups (white circles = CTL1, *n* = 10/white triangles = CTL2, *n* = 10); HF groups (black circles = HF1, *n* = 10/black triangles = HF2, *n* = 9). Asterisks indicate a significant difference between CTL and HF groups (*p < 0.05, **p ≤ 0.01, ***p ≤ 0.001). CTL, control; HF, high fat.

Mice receiving the HF diet during 12 weeks (HF1 and HF2) exhibited a significantly higher body weight (+23.0% and +22.3%, *p* < 0.05) (**Figure** **2**A,B) and AI (+157.0% and +152.0%, *p* < 0.05) (Figure 2C) compared to CTL mice (CTL1 and CTL2).

### 3.2 α‐Tocopherol Intake Is Not Different Between High‐Fat Diet‐Fed Mice and Control Mice

The mean food intake of mice under the CTL diet was significantly higher than that of mice under the HF diet (3.3 ± 0.5 g per mouse per day versus 2.2 ± 0.1 g per mouse per day, *p* = 0.02). According to the diet contents in α‐tocopherol (84 and 104 IU kg^−1^ for the CTL diet and HF diet, respectively), the total amount of α‐tocopherol consumed per day and per mouse was not significantly different between the two groups (0.28 ± 0.04 IU versus 0.23 ± 0.01 IU per mouse per day for CTL and HF diet, respectively, *p* = 0.1).

### 3.3 α‐Tocopherol Status Is Higher in High‐Fat Diet‐Fed Mice Than in Control Mice

**FIGURE 3:**
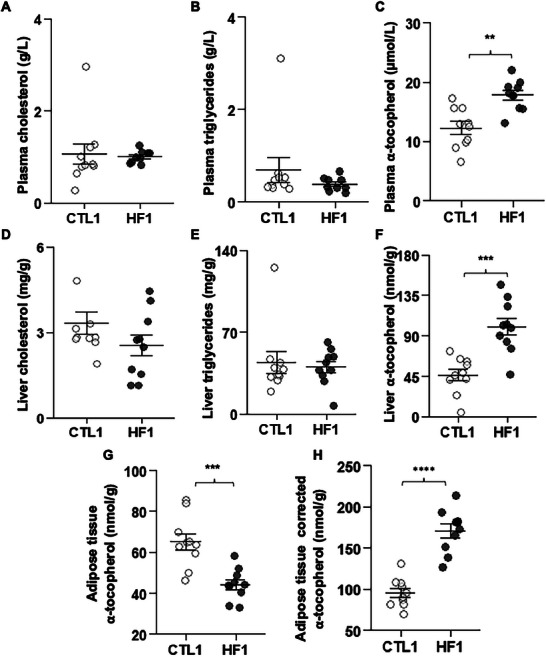
Plasma, liver, and adipose tissue lipid and α‐tocopherol concentration in fasting mice. Mice were fasted overnight before blood sampling. Lipids were assayed using biochemical assays, and α‐tocopherol was assayed by HPLC. A–c) Plasma cholesterol, triglycerides, and α‐tocopherol. d–f) Liver cholesterol, triglycerides, and α‐tocopherol. G,H) Adipose tissue α‐tocopherol and adipose tissue α‐tocopherol corrected for adiposity. White circles = CTL1, *n* = 10; black circles = HF1, *n* = 10. Asterisks indicate a significant difference between CTL and HF groups (*p < 0.05, **p ≤ 0.01, ***p ≤ 0.001, ****p ≤ 0.0001). CTL, control; HF, high fat.

No significant difference was found between CTL1 and HF1 groups regarding lipids in plasma and liver (**Figure** **3**A, B and D,E). However, α‐tocopherol concentration was higher in the plasma (Figure 3C) and in the liver of HF1 mice compared to CTL1 mice (Figure 3F, +45.0% and +116.9% in plasma and liver, respectively, *p* < 0.05).

AT α‐tocopherol concentration (Figure 3G) of the CTL1 group was higher than the HF1 group (+34.8%, *p* < 0.05). When α‐tocopherol concentration was corrected by the AI (α‐tocopherol concentration × AI, Figure 3H), α‐tocopherol concentration in the AT of HF1 group was higher than that of the CTL1 group (+72.4%, *p* < 0.05).

### 3.4 γ‐Tocopherol Absorption Is Increased in High‐Fat Diet‐Fed Mice Compared to Control Mice

**FIGURE 4:**
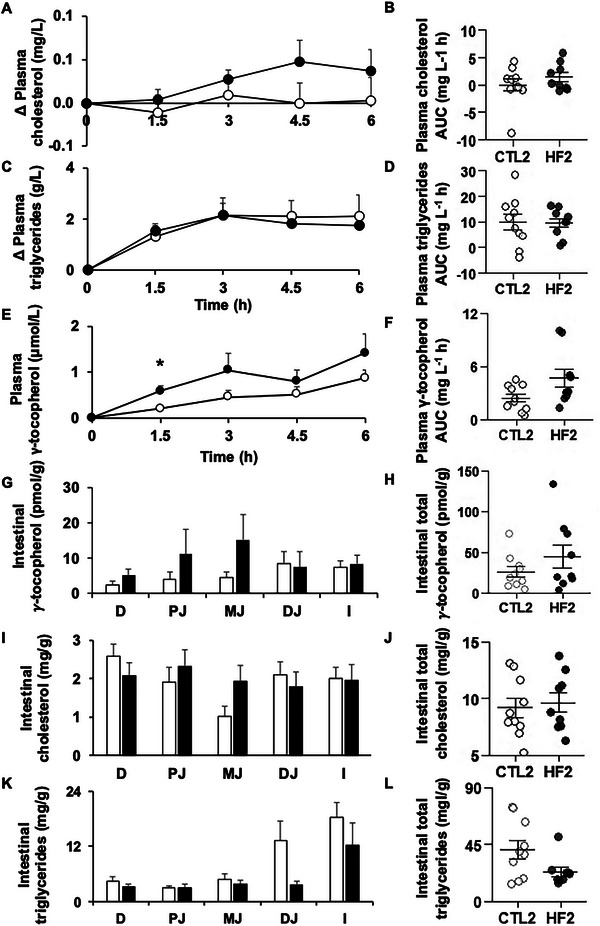
Lipid and γ‐tocopherol plasma responses and intestinal contents after force feeding. Mice were force‐fed with an oil‐in‐water emulsion containing γ‐tocopherol. Blood samples were taken every 1.5 h. Cholesterol and triglycerides were assayed using biochemical kits, and γ‐tocopherol was assayed by HPLC. A) Postprandial plasma cholesterol expressed as the difference between 1.5, 3, 4.5, 6, and 0 h. B) Area under the curve for plasma cholesterol. C) Postprandial plasma triglycerides expressed as the difference between 1.5, 3, 4.5, 6, and 0 h. D) Area under the curve for plasma triglycerides. E) Postprandial plasma γ‐tocopherol. F) Area under the curve for γ‐tocopherol. G) Intestinal content in cholesterol per segment. H) Total intestinal content in cholesterol. I) Intestinal content in triglycerides per segment. J) Total intestinal content in triglycerides. K) Intestinal content in γ‐tocopherol per segment. L) Total intestinal content in γ‐tocopherol. White circles/bars = CTL2. *n* = 10; black circles/bars = HF2, n = 9. Asterisks indicate a significant difference between CTL and HF groups (*p < 0.05, **p ≤ 0.01, ***p ≤ 0.001). CTL, control; D, duodenum; DJ, distal jejunum; HF, high fat; I, ileum; MJ, median jejunum; PJ, proximal jejunum.

Plasma cholesterol after force‐feeding did not change over time for CTL2 and had tendency to rise over time for HF2 but the difference remained nonsignificant (**Figure** **4**A,B). Following Triton injection, triglycerides accumulated in plasma as expected, and no differences were found between groups (Figure 4C,D).

No γ‐tocopherol was found in the plasma of fasting mice. γ‐Tocopherol response following force‐feeding was significantly higher in HF2 mice than in CTL2 mice 1.5 h after gavage (+181.5%, *p *< 0.05, Figure 4E). The plasma response in γ‐tocopherol, estimated with the area under the curve, was slightly higher for HF2 compared to CTL2 (+93.97%, *p* = 0.09, Figure 4F).

No significant difference was observed regarding cholesterol, triglyceride, and γ‐tocopherol accumulation in the intestine between HF and CLT groups (Figure 4G–L), although lipid and γ‐tocopherol amounts found in the intestine of HF mice tended to be higher than those found in the intestine of CTL mice.

### 3.5 Selected Intestinal Genes Are Not Differently Expressed in High‐Fat Diet‐Fed and Control Mice

**FIGURE 5:**
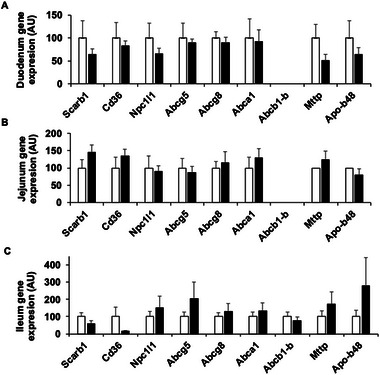
Gene expression in mouse intestine. A) Duodenum, B) jejunum, and C) ileum. White bars = CTL1, *n* = 10; black bars = HF1, *n* = 10. AU, arbitrary units; CTL, control; HF, high fat.

No significative difference was found between the CTL1 and HF1 groups for *Scarb1*, *Cd36*, *Npc1l1*, *Abcg5*, *Abcg8*, *Abca1*, *Abcb1b*, *Apo48*, and *Mttp* genes in any intestinal section (**Figure** **5**A–C).

## 4 Discussion

Lower plasma levels of fat‐soluble vitamins in patients with obesity compared to CTL patients have repeatedly been reported.^[^ ^2^, ^3^, ^4^, ^5^, ^6^, ^7^, ^8^ ^]^ Here, we specifically explored whether low VE status observed in patients with obesity could be due to a defect in absorption, using a rodent model with an overweight/obesity induced by a 3‐month HF diet.

When fed a HF diet, mice decrease their dietary intake to adjust their caloric consumption. This can lead to differences in terms of vitamin intake if vitamin amounts are not appropriately adjusted in the diets. α‐Tocopherol was the major form of VE in mouse diets. In this study, pellets used for HF groups of this study contained a higher α‐tocopherol concentration (104 IU kg^−1^) than pellets used for CTL groups (87 IU kg^−1^), which ensured similar α‐tocopherol intakes in both groups, in agreement with previous results.^[^ ^22^, ^23^ ^]^

We first assayed α‐tocopherol concentrations in plasma, liver, and AT of fasting mice to evaluate their VE status. HF mice had a higher plasma status in α‐tocopherol compared to CTL mice, while their triglyceride and cholesterol levels remained unchanged. α‐Tocopherol levels were thus still higher in the HF group compared to the CTL group when corrected by plasma lipids.^[^ ^24^ ^]^ This difference with results observed in human studies can be explained by the fact that α‐tocopherol intakes were controlled in our study. This may also be explained by a different α‐tocopherol metabolism between mice and humans, due to differences in lipoprotein synthesis and metabolism.^[^ ^25^ ^]^ HF mice also displayed higher α‐tocopherol amounts in liver and AT after correction by the AI than CTL mice. This last result indicates that in HF mice, α‐tocopherol is diluted in AT, but that HF mouse total α‐tocopherol stock is higher than that of CTL mice. A similar observation was reported for vitamin D in AT of HF mice.^[^ ^26^ ^]^ This result is consistent with a previous clinical study: β‐carotene concentration of human subcutaneous adipocytes was reduced in patients with obesity or with diabetes type 2 compared to CTLs.^[^ ^27^ ^]^ When data were corrected by total body fat, there was no difference between patients with obesity and those without obesity. Authors thus suggested that β‐carotene was diluted in the AT of patients with obesity or with diabetes type 2.^[^ ^27^ ^]^ This observation has also been indirectly confirmed in other studies. For instance, in a clinical trial, authors followed both BMI and plasma vitamin concentrations in untreated girls (CTL group) and in girls treated with valproic acid, because this treatment can cause obesity (*n* = 20 girls per groups).^[^ ^6^ ^]^ The girls who developed obesity after the treatment (*n* = 7) displayed low plasma levels in all vitamins. Once the treatment was completed, they returned to normal BMI and plasma vitamin levels. This study thus shows that vitamin status changes under obesity conditions, but that this can be reversible.^[^ ^6^ ^]^ Indeed, vitamin plasma status can be improved by losing weight, as demonstrated by a therapeutic program conducted in 103 adolescents aged 12–17 years old and diagnosed with overweight or obesity.^[^ ^28^ ^]^ In this study, both lipid‐corrected β‐carotene (*p* = 0.001) and α‐tocopherol (*p* < 0.001) plasma levels increased significantly after the program of weight loss. However, another study in boys (*n* = 70) and girls (*n* = 23) showed that after losing weight, eating less calories, and doing exercise, it was possible to restore carotenoid (β‐carotene) plasma levels, but not α‐tocopherol levels.^[^ ^29^ ^]^

We then explored both γ‐tocopherol and lipid postprandial responses in CTL and HF mice after an oral gavage with an oil‐in‐water emulsion enriched in γ‐tocopherol. γ‐Tocopherol was chosen over α‐tocopherol in this experiment for the following reasons. First, α‐and γ‐tocopherol are equally efficient at monitoring absorption because there is no discrimination between these forms at the intestinal level.^[^ ^30^, ^31^ ^]^ Second, both CTL and HF mice bred in‐house displayed undetectable concentrations of circulating γ‐tocopherol before gavage. Third, hepatic α‐tocopherol‐transfer protein resecretes γ‐tocopherol less efficiently than α‐tocopherol in VLDL. Taken together, these observations justify the use of postprandial plasma γ‐tocopherol as a natural tracer for newly absorbed VE.^[^ ^32^ ^]^

Plasma triglycerides after force‐feeding and Triton 1339 treatment reached a plateau as previously described.^[^ ^33^ ^]^ Such plateau is the consequence of the diminution of lipoprotein lipase activity: chylomicrons are not metabolized into remnants that can be taken up by the liver, so chylomicrons accumulate in the plasma. Cholesterol, which was not provided in the emulsion, lightly increased in the HF group after force‐feeding, which can be due to a concomitant VLDL accumulation in the plasma, a phenomenon also observed after a Triton treatment.^[^ ^33^ ^]^ γ‐Tocopherol response was higher in HF mice compared to CTL mice. Because chylomicron lipolysis was eliminated in this experiment, differences in terms of lipolysis and storage in an expanded AT can be excluded. Overall, this experiment shows that γ‐tocopherol is better absorbed in HF mice compared to CTL mice.

We hypothezized that this increase in γ‐tocopherol absorption could be due to differential protein expressions in the intestine of HF mice compared to CTLs. We thus studied the expression of genes encoding proteins involved in lipid and VE metabolism in mouse intestinal mucosa. *SRBI* and *NPC1L1* are key players in VE uptake. *SRBI, ABCG5*, and *ABCG8* can also likely facilitate VE efflux from the enterocyte to the lumen. *ABCA1* plays an important role in mediating the transport of VE via high‐density lipoprotein (HDL), at least in rodents, while *MTTP* and *APOB48* are involved in the assembly of chylomicrons within enterocytes.^[^ ^15^ ^]^ Our results showed that a HF diet did not significantly modulate the expression of any of these genes, at least in the fasting state. These results are partly consistent with a previous study showing that HF mice displayed similar expression levels of *Cd36* and *Npc1l1* compared to CTLs.^[^ ^34^ ^]^ This study also showed a decreased expression of *Scarb1* in HF mice^[^ ^34^ ^]^ but this result conflicts with prior results showing an increase in *Scarb1* expression – while other genes were not modulated – in mice under an HF diet.^[^ ^35^ ^]^ These discrepancies may be due to difference in protocols between studies (diet composition, period of exposure, fasting duration, etc.).

The reasons of such increased VE absorption and status in HF mice compared to CTL mice remain unknown. However, the HF diet markedly affects the gut bacterial ecosystem at the functional level^[^ ^36^ ^]^ and recent results have reported that intestinal microbiota can regulate mammalian lipid absorption, metabolism, and storage.^[^ ^37^ ^]^ It is therefore possible that the HF diet induces modifications in the microbiota of mice which influences their ability to absorb and store VE, at least in the early stage of obesity.

## 5 Conclusion

In our study, HF mice developed a moderate obesity and had the same VE intakes than CTL mice. HF mice exhibited both higher VE status and higher VE absorption efficiency than CTL mice. These results show that low VE status in patient with obesity is likely not due to an impairment in VE absorption, at least in the early stages of the disease. Further studies to determine whether obesity increases VE metabolism or excretion are therefore needed before advising any supplementation treatment in patients.

## Conflict of Interest

The authors did not declare any commercial or financial conflict of interest.

## Author Contributions

E.R. and J.F.L. designed the project. E.R. supervised this work. K.A.R., A.B.N., E.R., J.F.L., D.V., and C.S. conducted animal experiments. K.A.R., C.S., and D.V. did the biochemical analyses. K.A.R. and D.V. did the qPCR analyses. K.A.R. worked on the statistical analysis and data treatment. K.A.R. and E.R. wrote the manuscript, and K.A.R. prepared figures. All authors reviewed and approved the manuscript.

## Supporting information



Supporting Information

## Data Availability

The data that support the findings of this study are available from the corresponding author upon reasonable request.
