# Supplementary material for: Overweight Leads to an Increase in Vitamin E Absorption and Status in Mice
Source: Mol Nutr Food Res. 2024 Nov 16;68(23):2400509. doi: 10.1002/mnfr.202400509 (PMC11653163; doi:10.1002/mnfr.202400509)
Supplement: Supplementary file 1 — Supporting Information [file MNFR-68-2400509-s001.docx]

**Supplemental Table 1: Control and high-fat diet compositions**

|  | Control diet | High-fat diet |
| --- | --- | --- |
| Pregelatinized cornstarch (g/Kg) | 397.5 | 85 |
| Maltodextrin (g/Kg) | 142 | 115 |
| Sucrose (g/Kg) | 110 | 199.2 |
| Crude cellulose (g/Kg) | 50 | 58 |
| Casein (g/Kg) | 180 | 233 |
| L-cystein (g/Kg) | 3 | 3.5 |
| Soybean oil (g/Kg) | 70 | 30 |
| Lard (g/Kg) | 0 | 207 |
| Pre-mix of minerals PM AIN93M (g/Kg) | 35 | 35 |
| Pre-mix of vitamins PM AIN93M (g/Kg) | 10 | 12 |
| Choline bitartrate (g/Kg) | 2.5 | 2.3 |
| Potassium citrate (g/Kg) | 0 | 10 |
| Dicalcium phosphate (g/Kg) | 0 | 10 |
| Vitamin E (IU/Kg) | 87 | 104 |
| % Nitrogen Free Extract | 63.1 | 43.33 |
| % Starch | 33.79 | 7.23 |
| % Maltodextrin | 12.11 | 9.81 |
| % Total sugars | 13.83 | 22.17 |
| % Crude protein | 15.95 | 20.3 |
| % Crude fat | 7.39 | 24 |
| % Crude Ash | 2.7 | 4.04 |
| % Crude fiber | 3.57 | 4.08 |
| % Humidity | 7.29 | 4.55 |

From SAFE database

**Supplemental Table 2: ARN sequences used for qPCR analysis**

| **Gene** | **Acronym** | **ARN primer forward sequence** | **ARN primer reverse sequence** |
| --- | --- | --- | --- |
| **18s ribosomal ARN** | **18s** | CGCCGCTAGAGGTGAAATTCT | CATTCTTGGCAAATGCTTTCG |
| **Scavenger receptor class B type 1** | **SRB1** | CTGCCTAACATCTTGGTCCTG | AGCGCCAAGGTCATCATC |
| **CD36 molecule** | **CD36** | TTGTACCTATACTGTGGCTAAATGAGA | CTTGTGTTTTGAACATTTCTGCTT |
| **Niemann-Pick C1-Like 1** | **NPC1L1** | TACACGGCCTGGTCTTCCT | AGTACCAGAGCTTQGTTAACATC |
| **ATP-Binding Cassette subfamily G member 5** | **ABCG5** | TCCTGCATGTGTCCTACAGC | ATTTGCCTGTCCCACTTCTG |
| **ATP-Binding Cassette subfamily G member 8** | **ABCG8** | GAGCACTGTGCCTACGTCATCA | CGCAGGTTTGTCAGCCAGTA |
| **ATP-Binding Cassette subfamily A member 1** | **ABCA1** | TTCCTCAAAGTGGCTGAAGAG | TGCTGGCAAAGTACCATCTG |
| **ATP-Binding Cassette sub family B member 1b** | **ABCB1B** | AGTGGACCCAACAGTACTCTGAT | GCACCAATCCCGGTGTAATA |
| **Apolipoprotein B 48** | **APOB48** | CTGTTCAGTGGCAGCAACAC | 5’CAAGTTGACCAGGACTGCCT |
| **Microsomal Triglyceride Transfer Protein** | **MTTP** | AAGCCCCACTCAGGCAATTC | 5’CGGCCACTAGGCTCTCTTTT |
